# Tinnitus and COVID-19: effect of infection, vaccination, and the pandemic

**DOI:** 10.3389/fpubh.2024.1508607

**Published:** 2024-11-26

**Authors:** Yihsin Tai, Namitha Jain, Gibbeum Kim, Fatima T. Husain

**Affiliations:** ^1^Department of Speech Pathology and Audiology, Ball State University, Muncie, IN, United States; ^2^Department of Speech and Hearing Science, University of Illinois Urbana-Champaign, Champaign, IL, United States; ^3^Becknman Institute for Advanced Science and Technology, University of Illinois Urbana-Champaign, Urbana, IL, United States; ^4^Neuroscience Program, University of Illinois Urbana-Champaign, Urbana, IL, United States

**Keywords:** tinnitus, survey, hearing loss, COVID infection, long COVID, COVID vaccination

## Abstract

**Introduction:**

The COVID-19 pandemic, which began worldwide around March 2020, has had an impact on hearing health, specifically tinnitus and hearing loss. Physiologically, COVID-19 infection, or medication used to treat the infection, has been reported to be a potential risk factor for tinnitus onset. In addition, tinnitus was reported to be a long COVID symptom or to occur after a COVID-19 vaccination in some cases. With most reports focused on the clinical population, this study aimed to investigate how the onset of tinnitus is associated with COVID-19 infection, long COVID, and COVID-19 vaccination in the general population based on self-report, while accounting for otologic and psychological symptoms.

**Methods:**

In this study, a cross-sectional online survey that included general demographic questions, questions about tinnitus, hearing loss, hyperacusis, emotional status, and the Tinnitus Functional Index (TFI) was conducted.

**Results:**

Completed survey data of 1,511 respondents who reported having tinnitus or believed to have COVID-associated tinnitus were included in the analysis. Participants were categorized into four groups based on their judgment regarding the etiology of their tinnitus: (1) COVID infection group, (2) long COVID group, (3) COVID vaccination group, and (4) pre-existing tinnitus group. The results suggest that tinnitus severity (estimated using TFI scores) was significantly lower in the pre-existing tinnitus group than in any of the COVID-associated tinnitus groups. While varying factors were found to contribute to tinnitus severity among the COVID-associated groups, overall, depression and/or anxiety accounted for the most variance in predicting tinnitus severity.

**Discussion:**

The findings highlight the need to evaluate the impact of varying otologic and psychological symptoms in individuals with COVID-associated tinnitus for better patient-centered care.

## 1 Introduction

Since the COVID-19 pandemic began in early 2020, there have been reports of its impact on hearing health, particularly on the onset of tinnitus and/or hearing loss (1–3). These reports were initially case studies (e.g., (4, 5)) and later retrospective analyses of hospital data (e.g., (6, 7)). In these studies, otologic manifestations such as hearing loss, tinnitus, and vertigo were frequently reported during or after COVID-19 infection. There have also been reports suggesting that individuals who had tinnitus onset after COVID-19 infection showed similar tinnitus characteristics as those who had pre-existing tinnitus (e.g., (8)). The prevalence of tinnitus onset in COVID-19-infected individuals based on reviews of medical records displayed variability, ranging from 1.2 to 23.2% (3, 7, 9–11). These studies typically reported severe cases of COVID-19 infection; it was unclear often, if the sudden onset of hearing loss or tinnitus was due to the infection or the medications used to treat the infection (3, 8).

As the first wave of COVID-19 infection waned, a medical condition known as long COVID began to gain attention. This condition is characterized by patients who have recovered from the acute phases of the disease but failed to fully regain their prior levels of health and well-being, with symptoms that cannot be explained by other diagnoses persisting for at least 2 months (12). Initial investigations examining the association between COVID-19 and tinnitus onset did not account for this aspect. However, subsequent research revealed tinnitus as a probable symptom among individuals with long COVID (13–16). Nonetheless, available studies in this domain are limited and often display biases, relying primarily on observational designs within a confined number of countries. Thus, there is a need for a comprehensive global study to explore the intricate relationship between long COVID and its potential link to tinnitus.

The pandemic triggered intense global research efforts toward developing a vaccine against the disease. With the advent of the widely available messenger ribonucleic acid (mRNA) vaccines (e.g., Pfizer-BioNTech and Moderna) and the viral vector vaccines (e.g., AstraZeneca and Janssen), immediate side effects such as pain in the injection site, fever, fatigue, myalgia, and headache were commonly reported (17–20). However, later throughout the world, otologic symptoms such as sudden sensorineural hearing loss (SSNHL) and tinnitus began to be reported as adverse events following immunization (21). As a result, the Medicines and Healthcare products Regulatory Agency (MHRA) in the United Kingdom and the Vaccine Adverse Event Reporting System (VAERS) in the United States became popular sources for researchers to understand the recorded instances of tinnitus or SSNHL among recipients of COVID-19 vaccines, although these sources do not contain details of co-occurring otologic or psychological symptoms (22, 23). Additionally, reports from otologic clinics suggested an increasing number of visits due to complaints of otologic symptoms, with approximately 1.0–6.2% of patients reporting tinnitus after receiving COVID-19 vaccination (18, 24, 25). As vaccines became increasingly available and individuals who had recovered from COVID-19 infection also received vaccination, discerning the origin of symptoms like tinnitus became challenging (i.e., whether they were a result of the initial COVID-19 infection or the subsequent vaccination for it).

Apart from physiological changes due to the infection, vaccination, or medications used, many individuals have also reported increased experiences of social isolation, economic challenges, sleep disturbances, and psychological conditions such as depression and anxiety during the COVID-19 pandemic (26, 27). Such factors further contributed to tinnitus being more bothersome during the pandemic (26, 28, 29). While there is evidence suggesting worsened tinnitus during the pandemic, contrasting findings that suggested no significant changes in tinnitus before and after the pandemic in individuals with pre-existing tinnitus have also emerged (30, 31). Thus, there remains a need to understand the impact of the pandemic on pre-existing tinnitus, the onset of tinnitus, as well as the psychological sequelae.

Given the paucity of data on the impact of acute COVID-19 infection, long COVID, and COVID vaccination on tinnitus in the general population, we conducted an online survey targeting adults with tinnitus or who believed that their tinnitus was associated with COVID. Apart from asking about respondents’ tinnitus status and severity, we included a comprehensive set of questions regarding respondents’ physical and mental health. The aims of the study were (1) to investigate the prevalence of tinnitus believed to develop after COVID-19 infection, long COVID, or COVID vaccination (collectively termed as COVID-associated tinnitus throughout this paper), (2) to understand how otologic or psychological symptoms are linked with COVID-associated tinnitus or changes of tinnitus severity in pre-existing tinnitus, and (3) to investigate the difference in tinnitus severity among COVID-associated tinnitus, and how otologic or psychological symptoms affect tinnitus severity in COVID-associated tinnitus and pre-existing tinnitus.

## 2 Materials and methods

An online survey was conducted using the Qualtrics online software (Qualtrics Labs Inc., Provo, Utah, USA) between October 19, 2022, and August 29, 2023, under the University of Illinois Urbana-Champaign Institutional Review Board protocol number 23379. The link to the online survey was distributed through various channels that included e-mails, social media platforms, and charitable foundations associated with local, national, and international tinnitus support groups. Supplementary Table S1 provides an overview of the channels through which the survey was disseminated.

### 2.1 Participants

Adults above the age of 18 years who either had pre-existing tinnitus before the COVID-19 pandemic or had experienced tinnitus with an onset associated with COVID infection, long COVID, or COVID vaccination (even if their tinnitus had resolved by the time of filling out the survey) were eligible to take part in the study. Before the respondents began the survey, they were required to provide online informed consent. This consent process involved detailed descriptions of the study and sample questions related to the survey. All data collected during the study was anonymized, with no identifiable information being gathered from the respondents. Additionally, the geo-tracking feature of Qualtrics was disabled to protect participants’ privacy.

### 2.2 Measures

The survey questions were modified from online surveys used in previous studies in the authors’ lab (32, 33). During the development stage, a draft of the survey questions was reviewed by the authors, as well as several health professionals and researchers with tinnitus. The final survey consisted of 53 questions and the Tinnitus Functional Index (TFI: (34)) that required approximately 10–20 min to complete. The 53 questions included both closed-ended and open-ended formats, which were grouped with the TFI questions under four sections: (1) general demographic questions, (2) tinnitus-related questions, (3) hearing loss-related questions, and (4) comorbidities-related questions.

#### 2.2.1 General demographic questions

Questions in this section elicited information regarding age, gender, country of residence, and race/ethnicity. Individuals residing in China at the time of the survey were excluded, to comply with governmental restrictions within China. To ensure compliance with this exclusion, respondents were required to check a box confirming that they were not currently residing in China before they could begin the survey.

#### 2.2.2 Tinnitus-related questions

In this section, participants were first asked if they currently have tinnitus, with the answers being either “Yes” or “No, but I had tinnitus after having COVID infection, long COVID, or vaccination for COVID.” Participants who were currently experiencing tinnitus were asked to provide additional details regarding its duration, laterality, and the type of sound (ringing, buzzing, etc.). Tinnitus-related distress was measured using a rating scale for tinnitus annoyance and loudness. Participants rated the loudness of their tinnitus on a scale from 0 (very faint) to 10 (very loud), and tinnitus annoyance from 0 (not at all annoying) to 10 (extremely annoying). Additionally, the TFI, which comprises 25 questions across eight subsections (intrusive, sense of control, cognitive, sleep, auditory, relaxation, quality of life, and emotional), was utilized to evaluate the severity of respondents’ tinnitus.

Participants were also asked if their tinnitus onset was COVID-associated or was pre-existing before the pandemic. Based on their responses, they were categorized into four groups: (1) COVID infection group, (2) long COVID group, (3) COVID vaccination group, and (4) pre-existing tinnitus group. Respondents in COVID infection and long COVID groups were further asked about when they first noticed tinnitus symptoms, COVID test results, consultations with healthcare providers, treatments received, and the effectiveness of treatments for tinnitus. Those in the COVID vaccination group were asked about the onset of tinnitus symptoms after receiving vaccination, brand of vaccine, consultations with healthcare providers, treatments, and the effectiveness of treatments for tinnitus. Pre-existing tinnitus group respondents were asked about changes in their tinnitus during the pandemic, attributions for these changes, duration of changes, treatments sought, and the effectiveness of treatments for tinnitus. The follow-up questions for each group were meant to inform future thematic analysis; they were not used for data exclusion.

#### 2.2.3 Hearing loss-related questions

All participants were asked about the presence of hearing loss. If they indicated having hearing loss, follow-up questions about laterality, degree, duration, and the cause of their hearing loss were posed to gather more details. If participants attributed their hearing loss to COVID vaccination, additional follow-up questions were asked. These questions covered the specific brand of vaccine received and the onset of the hearing loss after vaccination.

#### 2.2.4 Comorbidities-related questions

All participants were asked specific questions related to hyperacusis, depression, and anxiety, as these are common co-morbidities with tinnitus. Hyperacusis status was determined if a participant selected the answer “Hypersensitivity to sounds (hyperacusis)” when answering the question “Which of the following do you currently experience?” Depression and anxiety were assessed using the Patient Health Questionnaire-4 (PHQ-4: (35)) depression and anxiety screener. The PHQ-4 consists of a two-item question for each condition, and participants scored each item from 0 to 3. The total sum scores for each condition range from 0 to 6. If a participant’s total sum score was greater than 2 for either the depression or anxiety sections, they were categorized as having depression or anxiety, respectively.

### 2.3 Statistical analysis

The survey data were analyzed using the R statistics software (version 4.2.1). Descriptive statistics, including the frequency and percentage of respondents’ demographic information were summarized in Table 1. Using data from questions that all respondents answered, various factors (including age, gender, hearing loss, hyperacusis, depression, and anxiety) were used for statistical analyses. To examine the difference in the aforementioned factors among groups, Chi-square tests followed by *post-hoc* pairwise proportion tests with Bonferroni correction were conducted for all factors, except for age. After checking the normality of age in each group using the Shapiro–Wilk test, Kruskal-Wallis rank sum test and *post-hoc* Dunn’s tests with Bonferroni correction for non-parametric data were used for age comparisons.

**Table 1:** Demographics of all respondents.

Demographics	*N* (of 1,511)	%
Gender		
Male or primarily masculine	723	47.85
Female or primarily feminine	782	51.75
Both male and female	1	0.06
Neither male nor female	3	0.2
Do not know	2	0.13
Age^a^		
18–25 years	28	1.85
26–40 years	215	14.23
41–55 years	443	29.32
56–70 years	596	39.44
>70 years	227	15.02
Unknown	2	0.13
Country		
United States	1,107	73.26
United Kingdom	106	7.02
Canada	62	4.1
Australia	50	3.31
New Zealand	37	2.45
Germany	21	1.39
Others^b^	128	8.47
Race/Ethnicity		
Caucasian	1,136	75.18
Asian	42	2.78
Hispanic	36	2.38
African American	11	0.73
Multiracial	10	0.66
Native American	3	0.2
Pacific Islander	0	0
Other	17	1.13
Prefer not to answer	19	1.26
Not applicable	237	15.68

a Age was stratified for the ease of presentation.

b Countries with less than 10 respondents.

To identify factors associated with increased risk of experiencing COVID-associated tinnitus, age- and gender-adjusted odds ratios (ORs) with 95% confidence intervals (CIs) were obtained using multivariate logistic regression. Similarly, in individuals with pre-existing tinnitus, age- and gender-adjusted ORs and 95% CIs were calculated using multivariate logistic regression to explore factors associated with increased risk of experiencing changes in tinnitus (tinnitus gets worse or better) after COVID infection, long COVID, or COVID vaccination.

To examine the difference in tinnitus severity (based on the TFI score) among the four groups, the Kruskal-Wallis rank sum test was used after confirming that the TFI scores were not normally distributed (*p* < 0.05 from the Shapiro–Wilk test of normality), followed by *post-hoc* Dunn’s tests with Bonferroni correction for multiple comparisons. Multiple linear regressions were then used to identify factors that significantly predict TFI scores in each group. Additionally, outliers and influential observations were examined, and sensitivity analyses were conducted to evaluate the robustness of the multiple linear regression models. All predictor variables included in the multivariate logistic regression and multiple linear regression models were dichotomous (e.g., male or female, having depression or not), except for age. The significance level was set at *p* < 0.05 for all statistical analyses.

Since this study focused on quantitative analyses using questions that most participants answered, details about individual respondents’ answers to open-ended questions or questions that provided an option for respondents to input their own answers (e.g., treatments tried) will be reported and analyzed in a different study using thematic analyses.

## 3 Results

### 3.1 Demographics

Table 1 shows the demographic data of the respondents who completed the survey. One thousand five hundred and eleven completed surveys were included in the data analysis. Most respondents were from the United States (73.26%), Caucasian (75.18%), and aged 41–55 years (29.32%) or 56–70 years (39.44%). Gender distribution was similar between males and females (around 50%).

#### 3.1.1 Group demographics and characteristics

Table 2 shows the demographics and characteristics among groups. Of the 1,511 respondents, 134 (8.87%), 136 (9%), 562 (37.19%), and 679 (44.94%) reported having tinnitus associated with COVID infection, long COVID, COVID vaccination, and unrelated to COVID (pre-existing tinnitus), respectively. Of those who reported COVID-associated tinnitus, eight respondents in the COVID infection group, five respondents in the long COVID group, and 39 respondents in the COVID vaccination group reported that they no longer had tinnitus, with the recovery rate ranging from 3.68 to 6.94%. The results of the Kruskal-Wallis test suggested a significant difference in median age among the groups (*H*(3) = 166.9, *p* < 0.001). *Post-hoc* Dunn’s tests with Bonferroni correction for multiple comparisons suggested that the pre-existing tinnitus group was significantly older in median age than any of the COVID-associated tinnitus groups. Among the four groups, the pre-existing tinnitus group had the highest proportion of male respondents (55.82%) and respondents who reported hearing loss (64.65%) but had the lowest rate of respondents who reported depression (18.7%) and anxiety (17.38%). The long COVID group had the highest rate of hyperacusis (54.41%), depression (33.09%), and anxiety (28.68%) among the four groups. Chi-square tests showed that the proportions of gender, hearing loss, hyperacusis, depression, and anxiety were significantly different among groups. *Post-hoc* pairwise proportion tests with Bonferroni correction showed that the pre-existing tinnitus group had significantly different proportions in gender, hearing loss, hyperacusis, depression, and anxiety compared with the long COVID and COVID vaccination groups. Additionally, the proportions in gender and hearing loss were significantly different between the COVID infection and pre-existing tinnitus groups. Notably, no significant differences in demographics and characteristics were found among the three COVID-associated tinnitus groups.

**Table 2:** Demographics, characteristics, and tinnitus severity across groups.

	Group				
Demographics	COVID infection (*n* = 134)	Long COVID (*n* = 136)	COVID vaccination (*n* = 562)	Pre-existing tinnitus (*n* = 679)	Test statistic
Gender	49 M, 84 F, 1 O	46 M, 90 F	249 M, 312 F, 1 O	379 M, 296 F, 4 O	*Χ*^2^ = 38.81***
Age in years: Mdn (IQR), range	54 (19), 18–76	52 (15), 18–73	53 (20), 19–87	63 (16), 18–94	*H* = 166.9***
Characteristic					
Tinnitus	126 (94.03)	131 (96.32)	523 (93.06)	679 (100)	-
Hearing loss	54 (40.3)	59 (43.38)	213 (37.9)	439 (64.65)	*Χ*^2^ = 98.44***
Hyperacusis	52 (38.81)	74 (54.41)	269 (47.86)	267 (39.32)	*Χ*^2^ = 16.88***
Depression	39 (29.1)	45 (33.09)	151 (26.87)	127 (18.7)	*Χ*^2^ = 21.07***
Anxiety	33 (24.63)	39 (28.68)	158 (28.11)	118 (17.38)	*Χ*^2^ = 23.07***
Tinnitus severity					
TFI score: Mdn (IQR)	50.8 (35.9)	60.8 (30.2)	56.4 (31.2)	42 (38)	*H* = 95.41***

Numbers for different characteristics are count and percentage (in parentheses).

F, female; IQR, interquartile range; M, male; Mdn, median; O, others; TFI, Tinnitus Functional Index.

***p < 0.001.

### 3.2 Relationship between tinnitus etiology and other factors

#### 3.2.1 COVID-associated tinnitus

**Figure 1:**
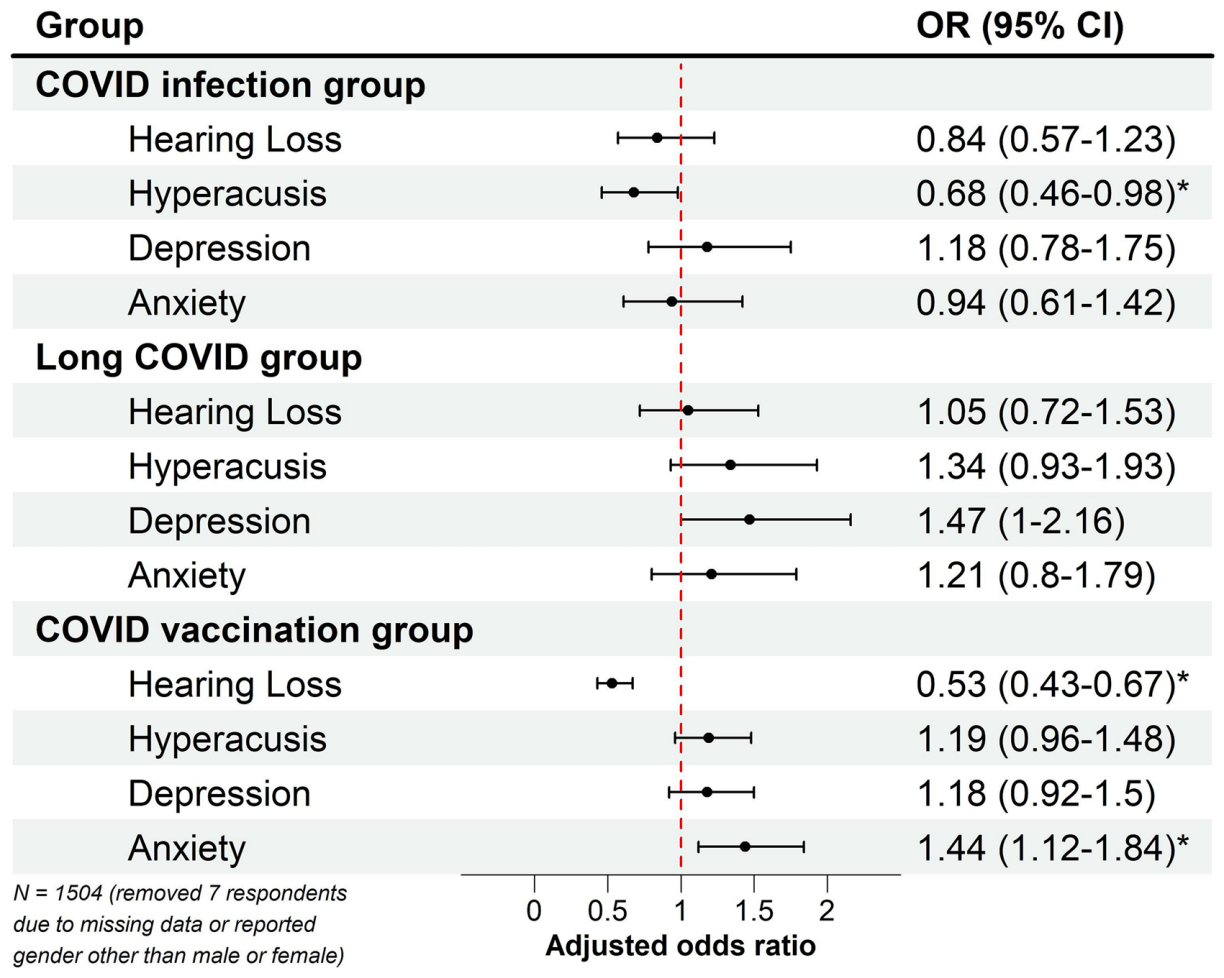
Forest plot showing the relationship between COVID-associated tinnitus and different otologic or psychological symptoms. Age- and gender-adjusted odds ratios (ORs) and 95% confidence intervals (CIs) were obtained using multivariate logistic regressions in each COVID-associated tinnitus group. The red dashed line denotes the reference line of no association between two examined factors (when ORs = 1). Significant associations were found between COVID infection-associated tinnitus and hyperacusis, as well as between COVID vaccination-associated tinnitus and hearing loss or anxiety. *p < 0.05.

Figure 1 shows the results of age- and gender-adjusted ORs and 95% CIs of each COVID-associated tinnitus group. Individuals who reported tinnitus associated with COVID infection had significantly decreased odds (OR 0.68; 95% CI 0.46–0.98) of reporting hyperacusis. The odds of reporting anxiety (OR 1.44; 95% CI 1.12–1.84) significantly increased in individuals who reported having tinnitus associated with COVID vaccination; however, these individuals had significantly decreased odds of reporting hearing loss (OR 0.53; 95% CI 0.43–0.67). Reporting tinnitus associated with long COVID did not significantly change the odds of reporting any of the examined factors.

#### 3.2.2 Changes in tinnitus in individuals with pre-existing tinnitus

Of the 679 respondents who reported having tinnitus before the COVID-19 pandemic, 294 (43.3%) reported changes in tinnitus (tinnitus gets worse = 283, 41.68%; tinnitus gets better = 11, 1.62%). Table 3 shows that none of the examined factors significantly increased or decreased the odds of reporting changes in pre-existing tinnitus following COVID infection, long COVID, or COVID vaccination.

**Table 3:** Age- and gender-adjusted odds ratios and 95% confidence intervals of changes in tinnitus following COVID-associated causes in those with pre-existing tinnitus.

Factor	Adjusted odds ratio (95% confidence interval)
Hearing loss	1.06 (0.62–1.82)
Hyperacusis	1.44 (0.87–2.4)
Depression	1.52 (0.83–2.91)
Anxiety	1.71 (0.93–3.29)

*n* = 294 (those who reported change in tinnitus in the pre-existing tinnitus group).

### 3.3 Tinnitus severity

**Figure 2:**
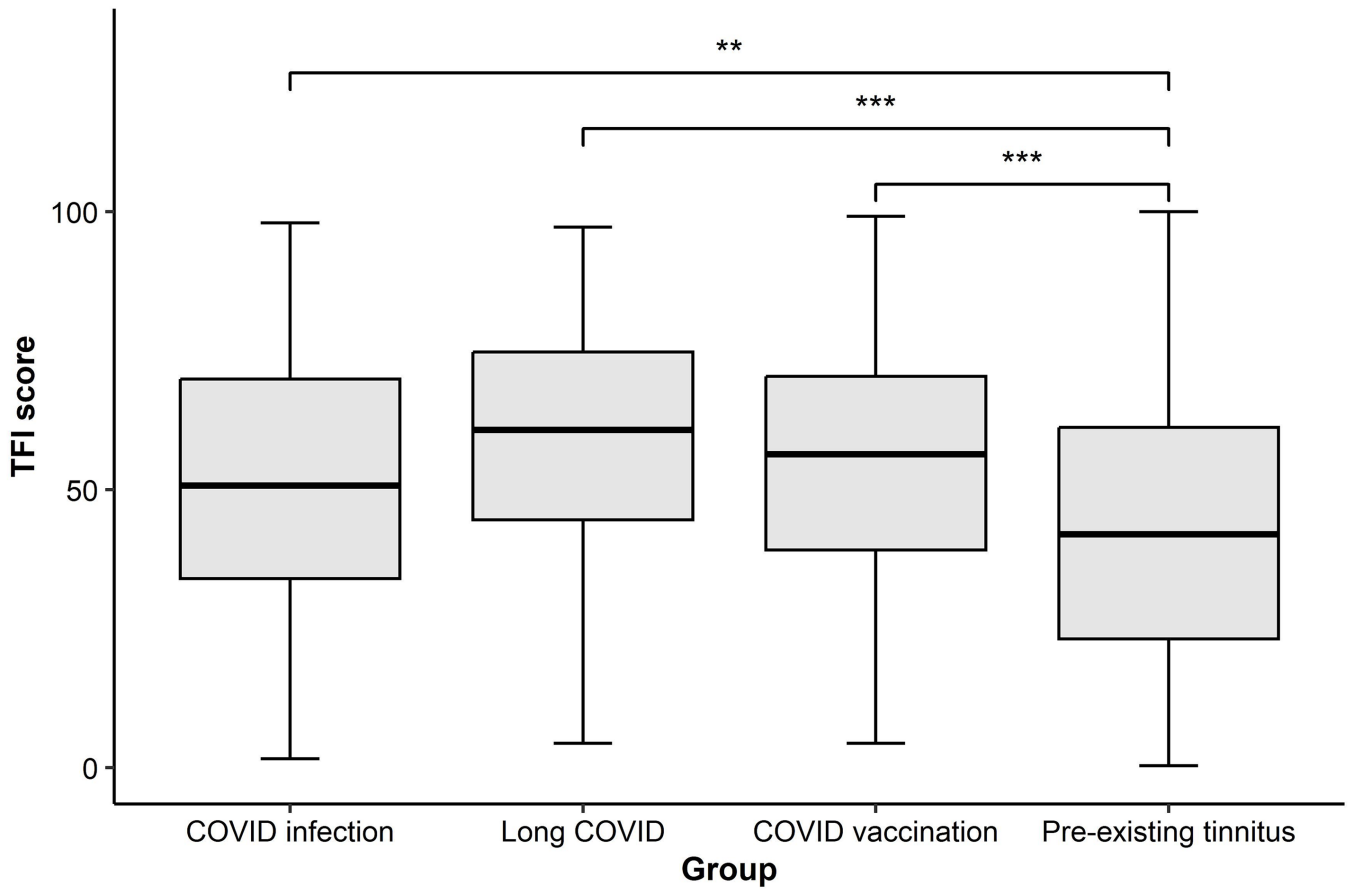
Boxplot showing the Tinnitus Functional Index (TFI) score in each group. The thick horizontal line within each box denotes the median value, and the box denotes the interquartile range. The error bars represent the maximum and minimum values of the TFI. The pre-existing tinnitus group showed a significantly lower median TFI score than any COVID-associated tinnitus group. **p < 0.01; ***p < 0.001.

As shown in Table 2, the median TFI score was the highest in the long COVID group (median = 60.8) and the lowest in respondents who had pre-existing tinnitus (median = 42). The results of the Kruskal-Wallis rank sum test suggest that median TFI scores were significantly different among groups (*H*(3) = 95.41, *p* < 0.001). *Post-hoc* Dunn’s tests with Bonferroni correction for multiple comparisons suggest that all three COVID-associated tinnitus groups had significantly higher median TFI scores compared with the pre-existing tinnitus group (Figure 2).

#### 3.3.1 Factors related to tinnitus severity

The results of the multiple linear regression analyses are reported in Table 4, which shows the factors contributing to the increase in tinnitus severity (heightened TFI score) in each group. It should be noted that the results in Table 4 were obtained on a reduced sample size of each COVID-associated tinnitus group, as some respondents reported not having tinnitus at the time of answering the survey questions (see Table 2). In the COVID infection group, the overall regression was statistically significant, with the model explaining 27.1% of the variance (*R*^2^ = 0.271, *F*(6, 118) = 8.67, *p* < 0.001). However, gender, hearing loss, and anxiety were found to be the only significant predictors of the TFI scores. In the long COVID group, the model explained 27.9% of the variance (*R*^2^ = 0.279, *F*(6, 124) = 9.4, *p* < 0.001), but only hyperacusis and depression significantly predicted the TFI scores. In both the COVID vaccination and pre-existing tinnitus groups, age, hearing loss, hyperacusis, depression, and anxiety were all found to be significant predictors of the TFI scores. However, gender was also a significant predictor of the TFI scores in the pre-existing tinnitus group. The overall regressions were statistically significant in those two groups, with the models explaining 26.1% of the variance in the COVID vaccination group (*R*^2^ = 0.261, *F*(6, 516) = 31.78, *p* < 0.001), and 25.6% of the variance in the pre-existing tinnitus group (*R*^2^ = 0.256, *F*(6, 665) = 39.41, *p* < 0.001). The results of the sensitivity analyses showed that the effects of most predictor variables did not change significantly after removing outliers and influential observations from the models (see Supplementary Table S2). However, the variances explained in the models of the COVID infection and long COVID groups increased to >45%, suggesting the robustness of the models might be affected in groups with small sample sizes.

**Table 4:** Multiple linear regression models to predict the Tinnitus Functional Index (TFI) score in different groups.

	COVID infection group (*n* = 125)		Long COVID group (*n* = 131)		COVID vaccination group (*n* = 523)		Pre-existing tinnitus group (*n* = 674)	
Variables	*β*	*p*	*β*	*p*	*β*	*p*	*β*	*p*
Age	0.072	0.381	0.079	0.331	0.128	**<0.001**	0.076	**0.034**
Gender (Male)	−0.225	**0.006**	−0.042	0.595	−0.002	0.969	−0.067	**0.048**
Hearing loss (Yes)	0.193	**0.019**	0.142	0.067	0.115	**0.003**	0.169	**<0.001**
Hyperacusis (Yes)	−0.022	0.79	0.19	**0.019**	0.099	**0.012**	0.15	**<0.001**
Depression (Yes)	0.133	0.241	0.438	**<0.001**	0.273	**<0.001**	0.276	**<0.001**
Anxiety (Yes)	0.352	**0.002**	0.074	0.49	0.255	**<0.001**	0.214	**<0.001**
*R* ^2^	0.271		0.279		0.261		0.256	

*β, Standardized beta.*

Bold numbers indicate statistical significance.

## 4 Discussion

To better understand otologic and psychological symptoms reported in COVID-associated tinnitus and pre-existing tinnitus, we conducted an online survey targeting the general population. The respondents were divided into three groups based on their judgment regarding tinnitus onset – after COVID infection, due to long COVID, or occurred soon after a COVID vaccination. A fourth group with pre-existing tinnitus was also included. Our findings showed that the rates of hearing loss, hyperacusis, depression, and anxiety were significantly different between COVID-associated tinnitus groups and the pre-existing tinnitus group. The findings also indicated that the COVID infection group had significantly lower odds of reporting hyperacusis and that the COVID vaccination group had significantly lower odds of reporting hearing loss. However, the COVID vaccination group showed increased odds of reporting anxiety. In individuals with pre-existing tinnitus, we did not find significant associations between any factor and the reported COVID-related changes in tinnitus severity. Overall, tinnitus severity was significantly higher in any of the COVID-associated tinnitus groups compared with the pre-existing tinnitus group, with several predictors, particularly depression and/or anxiety, accounting for the most variance.

### 4.1 Factors related to COVID-associated tinnitus

Upon further examination of each factor related to COVID-associated tinnitus, we found that the COVID infection group had significantly lower odds of reporting hyperacusis, after accounting for age and gender. While hyperacusis has been suggested as one potential otologic symptom after COVID infection (36), there has been no clear evidence supporting increased co-occurrence of tinnitus and hyperacusis after COVID infection (6). Notably, the COVID infection group also reported the lowest rate (38.81%) of hyperacusis among all groups.

Although symptoms identified in adults with long COVID include depression, anxiety, hearing loss, or tinnitus (37–40), we did not find a significant relation among long COVID-associated tinnitus and any aforementioned factors. This non-significant finding may be due to reduced statistical power from a small sample size. Presently, the prevalence of long COVID-associated tinnitus in the general population is still unclear; the smaller sample size in the long COVID group in comparison to the COVID vaccination group in our study may simply reflect that long COVID-associated tinnitus is relatively uncommon (38, 39) or fewer such participants chose to complete the survey. In a large-scale study on individuals who had a COVID diagnosis (41), the key symptoms of COVID-19 sequelae over time (ranging from 2 to 18 months) included fatigue, dyspnea, and neuropsychiatric symptoms such as brain fog; however, tinnitus was found to be an unlikely reported symptom. Moreover, without accounting for potential risk factors of long COVID such as having multiple and/or severe symptoms during the initial COVID-19 infection (41–43), a strong correlation between long COVID-associated tinnitus and other factors cannot be easily drawn.

Growing evidence suggests that adverse events following COVID vaccination include tinnitus, SSNHL, and/or vestibular symptoms (21); however, the risk of new-onset tinnitus or SSNHL due to COVID vaccination is considerably lower than the risk caused by other commonly adult-administered vaccines, such as Tdap or influenza vaccines (22, 23, 44, 45). A recent review indicates that SSNHL after COVID vaccination was mostly associated with tinnitus, probably due to sensory deprivation caused by inner ear damage following COVID vaccination (21). Similarly, Leong et al. (18) found that in a group of 420 vaccinated patients, SSNHL following COVID vaccination mostly co-occurred with tinnitus. However, our findings suggested that in individuals who reported COVID vaccination-associated tinnitus, the odds of reporting hearing loss were significantly decreased. The contradictory findings between our study and previous studies may stem from the difference in the targeted populations. While most previous studies focused on patients who sought medical attention after experiencing SSNHL following COVID vaccination (18, 21, 22, 25), our survey might capture the responses that better represent the general population, with most respondents reporting minimal concern about hearing loss that entails medical attention. Nonetheless, an assessment of the sudden nature of hearing loss is needed to further delineate the association between hearing loss and tinnitus in the COVID vaccination group.

Despite ample support from the literature on the relationship between anxiety and tinnitus (46, 47), only individuals with COVID vaccination-associated tinnitus had significantly increased odds of reporting anxiety. While the exact mechanism of COVID vaccine-induced tinnitus remains unclear (44), it has been proposed that adverse events to vaccines may not only be triggered by the ingredients in the vaccine but also be contributable to the stress or anxiety throughout the vaccination process and the global pandemic that necessitated said vaccination (48, 49). An increased fear and anxiety relating to the COVID vaccination has been identified in the general population ever since the COVID vaccines were made public (50). However, anxiety-related adverse events can often be underestimated. With the uncertainty about the long-term effects of new vaccines, negative expectations about the COVID vaccines (a.k.a. a nocebo effect) that widely spread through various informational sources may aggregate the reports of anxiety-related adverse effects (48). The nocebo effect demonstrates the critical role of hypothetical health beliefs in the increased likelihood of physical sensations. Such effect can be inextricably linked to events or symptoms reported during the pandemic, as evident in individuals with particular beliefs about COVID-19 later reporting increased auditory symptoms or experiencing unexplained bodily symptoms (51, 52). Although the significant relation between tinnitus and anxiety found in our COVID vaccination group may support the notion of anxiety-related adverse events during the vaccination process, our data were insufficient to establish a causality link.

### 4.2 Factors related to changes in tinnitus

The impact of the pandemic on pre-existing tinnitus has been investigated in several previous studies (e.g., (30)). Our results on the changes in tinnitus severity in pre-existing tinnitus moderately echo the Beukes et al. (26) study, which shows that 32% of respondents reported their tinnitus being more bothersome during the pandemic, with only 1% reporting improvement of tinnitus. On the contrary, one survey study targeting older adults with tinnitus showed that only 10% of their respondents reported changes in their perception of tinnitus severity after the pandemic (31). Notably, heterogeneity across studies was also seen in studies that compared the scores of the Tinnitus Handicap Inventory (THI: (53)) or visual analog scale (VAS) of annoyance between pre- and peri-pandemic: significantly increased tinnitus severity (manifested by higher THI or VAS scores) during the pandemic was found in some studies (27–29) but not others (30). One point of difference is that our survey was conducted in late 2022 and early 2023, when the general public had likely habituated to the idea of the pandemic. Regardless of the mixed findings, most studies acknowledged the inextricable link between emotional states and tinnitus, with findings suggesting a significant association between increased anxiety and/or depression during the pandemic and increased tinnitus severity (27–29).

### 4.3 Tinnitus severity among groups

Based on the TFI scores, tinnitus severity was significantly greater in any of the COVID-associated tinnitus groups relative to the pre-existing tinnitus group. Our findings support the negative association between tinnitus duration and tinnitus severity (54), as individuals in COVID-associated tinnitus groups had relatively shorter tinnitus duration compared with those in the pre-existing tinnitus group. Another possible explanation lies in the fact that the characteristics observed in COVID-associated tinnitus groups were different from those observed in the pre-existing tinnitus group. In our respondents, the pre-existing tinnitus group showed significantly different rates of hearing loss, hyperacusis, depression, and anxiety compared with other groups. The higher rate of reported hearing loss in the pre-existing tinnitus group, which had a higher proportion of males and significantly older respondents compared with other groups, is not surprising, considering the known association among age, gender, and the likelihood of tinnitus (55). However, among all groups, the rates of reporting hyperacusis, depression, and anxiety were lower in the pre-existing tinnitus group. This agrees with reports on the potential effect of tinnitus duration on the level of depression and anxiety (56). Although there was no significant difference in tinnitus severity among the COVID-associated tinnitus groups, our findings suggest that the long COVID group reported the highest median TFI score. While a comprehensive understanding of the physiological or psychological mechanisms of long COVID-related tinnitus is emerging, growing evidence suggests that long COVID can have a debilitating impact on an individual (37, 57). With long COVID being recognized as a disability according to the guidance of the Americans with Disabilities Act (58), it may not be surprising that individuals who experience several symptoms of long COVID across multiple organs may perceive greater tinnitus severity compared to those without long COVID.

Further analysis suggested that only around 26–27% of the variance in tinnitus severity (TFI scores) was explained by the factors we examined. The low variances may be because the factors included do not necessarily correspond to several subscales probed by the TFI, such as the intrusive or sleep subscales (34). Moreover, other factors that might affect tinnitus severity, such as loudness of tinnitus, personality traits, and survey environment (46, 59, 60) were not included. Overall, depression and/or anxiety contributed the most to the variance of the TFI scores in all groups. The findings are not unpredictable considering the strong link between emotional states and tinnitus severity (47, 61). However, significant predictors in the COVID infection and long COVID groups only contain one of the emotional states. This suggests that in these two groups, individuals may find their tinnitus bothersome even without the co-occurring depression and anxiety. It is noteworthy that the models for the COVID vaccination group and the pre-existing tinnitus group shared identical predictors except for gender. While the models for both groups shared anxiety and depression as the most significant predictors of tinnitus severity, the overall tinnitus severity is significantly higher in the COVID vaccination than in the pre-existing tinnitus group. With the potential additive effect of co-occurring anxiety and depression on tinnitus distress (62), one possible explanation is that such an additive effect may have a greater impact on tinnitus severity in the COVID vaccination group than in the pre-existing tinnitus group. Together, our findings highlight the importance of considering different aspects, especially psychological ones, while assessing tinnitus severity in individuals with varying COVID-associated onset of tinnitus.

### 4.4 Limitations and future directions

Several limitations should be noted. First, like many tinnitus survey studies (see the review by Jarach et al. (63)), only a general definition of tinnitus (ringing, buzzing, or noise in the ear) was used in our survey. Without including questions or assessments to identify subtypes of tinnitus such as somatosensory tinnitus (64), our findings may oversimplify the relationship between COVID-19 and tinnitus. Second, biases such as sample bias and recall bias can be introduced depending on the survey questions and how the survey was disseminated. In regards to sample bias, since only individuals who received the survey link could complete the survey, and the decision to complete the survey may be driven by varying factors (being more technologically inclined, wanting to learn more about the study, having a higher level of anxiety or depression, wanting to seek support for their tinnitus, etc.), the responses we received might not be fully representative. The unequal sample sizes among groups in our study could also affect the statistical power. This is usually a problem when targeting multiple groups in one survey study, with our recruitment message possibly drawing more respondents in one group than another. Additionally, our data are sensitive to recall bias. If the onset of tinnitus was directly associated with a COVID-related cause, the tinnitus onset should be similar to that of symptoms observed after COVID infection, long COVID, or COVID vaccination (22, 24, 65). However, considering that our respondents might have experienced multiple COVID-related symptoms when completing the survey, the respondents might link their tinnitus to a specific COVID-associated event when they were unable to remember previous events accurately. As such, even though many studies have attempted to unfold the association between tinnitus and COVID-19, the knowledge gap could still be enlarged due to inconsistent reporting, recall bias, or the nocebo effect (52, 65). Third, to reduce survey length, we were unable to include validated questionnaires to evaluate every factor, such as hyperacusis and hearing loss. Even though the PHQ-4 was used to assess the respondents’ risk of having anxiety and depression, the accuracy of the assessment may not be comparable to more comprehensive questionnaires. Therefore, our findings, which were largely based on the analyses of responses to single questions, should be interpreted with caution. Fourth, as the study was cross-sectional, the causal relationship between different factors cannot be established. Moreover, we were unable to evaluate the long-term effect of different COVID-associated causes on tinnitus. Lastly, as emerging evidence suggests hearing disorders such as tinnitus may result from neuroinflammation responding to varying diseases (66), one unanswered question of our study is: what physiological mechanism(s) causes a higher incidence of tinnitus in individuals who received immunization or suffered from COVID relative to those who did not undergo these conditions? Future longitudinal studies that include control groups and comprehensive audiological and physiological assessments are warranted to better understand the causality of COVID-associated factors and tinnitus.

## 5 Conclusion

Our findings suggest that several factors, such as hearing loss or hyperacusis, were significantly correlated with the report of tinnitus in individuals who attributed their tinnitus to varying COVID-associated causes. The results highlight the significant effects of anxiety and/or depression on tinnitus severity, regardless of the reported cause of tinnitus. Not surprisingly, cognitive-behavioral therapy or mindfulness-based stress reduction has often been recommended for reducing tinnitus distress in individuals with COVID-associated tinnitus (26–28). Our findings also underline the importance of incorporating clinical assessments to better estimate the impact of varying hearing and psychological disorders on tinnitus severity in those with COVID-associated tinnitus. With COVID infection, long COVID, and COVID vaccination becoming potential causes of tinnitus, more individualized clinical management that accounts for relevant factors may be required to provide patient-centered care. Although evidence-based research focused on intervention in COVID-associated tinnitus is still lacking, with the complex nature of COVID-related symptoms, it is evident that a multidisciplinary approach including professionals such as audiologists, otolaryngologists, psychologists, and neurologists is essential to address potential challenges in this tinnitus population (67).

## Data Availability

The raw data supporting the conclusions of this article will be made available by the authors, without undue reservation.

## References

[1] Karimi-Galougahi M Naeini AS Raad N Mikaniki N Ghorbani J. Vertigo and hearing loss during the covid-19 pandemic – is there an association? Acta Otorhinolaryngol Ital. (2020) 40:463–5. doi: 10.14639/0392-100X-N0820, PMID: 32519994 PMC7889249

[2] Koumpa FS Forde CT Manjaly JG. Sudden irreversible hearing loss post COVID-19. BMJ Case Rep. (2020) 13:e238419. doi: 10.1136/bcr-2020-238419, PMID: 33051251 PMC7554505

[3] Munro KJ Uus K Almufarrij I Chaudhuri N Yioe V. Persistent self-reported changes in hearing and tinnitus in post-hospitalisation COVID-19 cases. Int J Audiol. (2020) 59:889–90. doi: 10.1080/14992027.2020.1798519, PMID: 32735466

[4] Lamounier P Gonçalves VF Ramos HVL Gobbo DA Teixeira RP Dos Reis PC. A 67-year-old woman with sudden hearing loss associated with SARS-CoV-2 infection. Am J Case Rep. (2020) 21:1–6. doi: 10.12659/AJCR.927519, PMID: 33139689 PMC7650213

[5] Parrino D Frosolini A Gallo C De Siati RD Spinato G de Filippis C. Tinnitus following COVID-19 vaccination: report of three cases. Int J Audiol. (2022) 61:526–9. doi: 10.1080/14992027.2021.1931969, PMID: 34120553

[6] AlJasser A Alkeridy W Munro KJ Plack CJ. Is COVID-19 associated with self-reported audio-vestibular symptoms? Int J Audiol. (2022) 61:832–40. doi: 10.1080/14992027.2021.1957161, PMID: 34370603

[7] Gallus R Melis A Rizzo D Piras A De Luca LM Tramaloni P. Audiovestibular symptoms and sequelae in COVID-19 patients. J Vestib Re. (2021) 31:381–7. doi: 10.3233/VES-201505 33579886

[8] Figueiredo RR Penido NO Azevedo AA Oliveira PM Siqueira AG Figueiredo GMR. Tinnitus emerging in the context of a COVID-19 infection seems not to differ in its characteristics from tinnitus unrelated to COVID-19. Front Neurol. (2022) 13:974179. doi: 10.3389/fneur.2022.974179, PMID: 36158941 PMC9505692

[9] Zięba N Lisowska G Dadok A Kaczmarek J Stryjewska-makuch G Misiołek M. Frequency and severity of ear–nose–throat (ENT) symptoms during COVID-19 infection. Medicina. (2022) 58:623. doi: 10.3390/medicina58050623, PMID: 35630040 PMC9143391

[10] Elibol E. Otolaryngological symptoms in COVID-19. Eur Arch Otorrinolaringol. (2021) 278:1233–6. doi: 10.1007/s00405-020-06319-7, PMID: 32875391 PMC7461752

[11] Viola P Ralli M Pisani D Malanga D Sculco D Messina L. Tinnitus and equilibrium disorders in COVID-19 patients: preliminary results. Eur Arch Otorrinolaringol. (2021) 278:3725–30. doi: 10.1007/s00405-020-06440-7, PMID: 33095432 PMC7582442

[12] Soriano JB Murthy S Marshall JC Relan P Diaz JV. A clinical case definition of post-COVID-19 condition by a Delphi consensus. Lancet Infect Dis. (2022) 22:e102–7. doi: 10.1016/S1473-3099(21)00703-9, PMID: 34951953 PMC8691845

[13] Berlot AA Moskowitz HS Lin J Liu J Sehanobish E Jerschow E. Acute and longer-term effects of COVID-19 on auditory and vestibular symptoms. Otol Neurotol. (2023) 44:1100–5. doi: 10.1097/MAO.0000000000004027, PMID: 37758317

[14] Degen CV Mikuteit M Niewolik J Schröder D Vahldiek K Mücke U. Self-reported tinnitus and vertigo or dizziness in a cohort of adult long COVID patients. Front Neurol. (2022) 13:884002. doi: 10.3389/fneur.2022.884002, PMID: 35547372 PMC9082801

[15] Dorobisz K Pazdro-Zastawny K Misiak P Kruk-Krzemień A Zatoński T. Sensorineural hearing loss in patients with long-COVID-19: objective and behavioral audiometric findings. Infect Drug Resist. (2023) 16:1931–9. doi: 10.2147/IDR.S398126, PMID: 37025195 PMC10072149

[16] Haider HF Szczepek AJ. Editorial: Neurotological consequences of long COVID. Front Neurol. (2022) 13:1087896. doi: 10.3389/fneur.2022.1087896, PMID: 36479046 PMC9720380

[17] Baden LR El Sahly HM Essink B Kotloff K Frey S Novak R. Efficacy and safety of the mRNA-1273 SARS-CoV-2 vaccine. N Engl J Med. (2021) 384:403–16. doi: 10.1056/nejmoa2035389, PMID: 33378609 PMC7787219

[18] Leong S Teh BM Kim AH. Characterization of otologic symptoms appearing after COVID-19 vaccination. Am J Otolaryngol. (2023) 44:103725. doi: 10.1016/j.amjoto.2022.103725, PMID: 36525812 PMC9721195

[19] Polack FP Thomas SJ Kitchin N Absalon J Gurtman A Lockhart S. Safety and efficacy of the BNT162b2 mRNA Covid-19 vaccine. N Engl J Med. (2020) 383:2603–15. doi: 10.1056/nejmoa2034577, PMID: 33301246 PMC7745181

[20] Sadoff J Gray G Vandebosch A Cárdenas V Shukarev G Grinsztejn B. Safety and efficacy of single-dose Ad26.COV2.S vaccine against Covid-19. N Engl J Med. (2021) 384:2187–201. doi: 10.1056/nejmoa2101544, PMID: 33882225 PMC8220996

[21] Pisani D Gioacchini FM Viola P Scarpa A Astorina A Re M. Audiovestibular disorders after COVID-19 vaccine: is there an association? Audiol Res. (2022) 12:212–23. doi: 10.3390/audiolres12030024, PMID: 35645194 PMC9149883

[22] Formeister EJ Wu MJ Chari DA Meek R Rauch SD Remenschneider AK. Assessment of sudden sensorineural hearing loss after COVID-19 vaccination. JAMA Otolaryngol Head Neck Surg. (2022) 148:307–15. doi: 10.1001/jamaoto.2021.4414, PMID: 35201274 PMC8874871

[23] Yih WK Duffy J Su JR Bazel S Fireman B Hurley L. Tinnitus after COVID-19 vaccination: findings from the vaccine adverse event reporting system and the vaccine safety datalink. Am J Otolaryngol. (2024) 45:104448. doi: 10.1016/j.amjoto.2024.104448, PMID: 39096568 PMC11634645

[24] Lin D Selleck AM. Tinnitus cases after COVID-19 vaccine administration, one institution’s observations. Am J Otolaryngol. (2023) 44:103863. doi: 10.1016/j.amjoto.2023.103863, PMID: 36989754 PMC10036149

[25] Wichova H Miller ME Derebery MJ. Otologic manifestations after COVID-19 vaccination: the House ear clinic experience. Otol Neurotol. (2021) 42:e1213–8. doi: 10.1097/MAO.0000000000003275, PMID: 34267103 PMC8443418

[26] Beukes EW Baguley DM Jacquemin L Lourenco MPCG Allen PM Onozuka J. Changes in tinnitus experiences during the COVID-19 pandemic. Front Public Health. (2020) 8:592878. doi: 10.3389/fpubh.2020.592878, PMID: 33251179 PMC7676491

[27] Schlee W Hølleland S Bulla J Simoes J Neff P Schoisswohl S. The effect of environmental stressors on tinnitus: a prospective longitudinal study on the impact of the covid-19 pandemic. J Clin Med. (2020) 9:2756. doi: 10.3390/jcm9092756, PMID: 32858835 PMC7565885

[28] Aydoǧan Z Çinar Satekin M Ocak E Tokgoz Yilmaz S. Effects of the coronavirus disease 2019 pandemic on subjective tinnitus perception. J Laryngol Otol. (2022) 136:410–3. doi: 10.1017/S0022215122000640, PMID: 35510491 PMC8961067

[29] Xia L He G Feng Y Yu X Zhao X Yin S. COVID-19 associated anxiety enhances tinnitus. PLoS One. (2021) 16:e0246328. doi: 10.1371/journal.pone.0246328, PMID: 33544744 PMC7864409

[30] Aazh H Danesh AA Moore BCJ. Self-reported tinnitus severity prior to and during the COVID-19 lockdown in the United Kingdom. J Am Acad Audiol. (2021) 32:562–6. doi: 10.1055/s-0041-1731733, PMID: 35176799

[31] Jarach CM Lugo A Stival C Bosetti C Amerio A Cavalieri d’Oro L. The impact of COVID-19 confinement on tinnitus and hearing loss in older adults: data from the LOST in Lombardia study. Front Neurol. (2022) 13:366. doi: 10.3389/fneur.2022.838291, PMID: 35330807 PMC8940241

[32] Husain FT Chappell J Tai Y. An online survey study of the association between tinnitus and hyperacusis using validated questionnaires. Int J Audiol. (2022) 61:655–62. doi: 10.1080/14992027.2021.1953712, PMID: 34353201

[33] Husain FT Gander PE Jansen JN Shen S. Expectations for tinnitus treatment and outcomes: a survey study of audiologists and patients. J Am Acad Audiol. (2018) 29:313–36. doi: 10.3766/jaaa.16154, PMID: 29664725

[34] Meikle MB Henry JA Griest SE Stewart BJ Abrams HB McArdle R. The tinnitus functional index: development of a new clinical measure for chronic, intrusive tinnitus. Ear Hear. (2012) 33:153–76. doi: 10.1097/AUD.0b013e31822f67c0 22156949

[35] Löwe B Wahl I Rose M Spitzer C Glaesmer H Wingenfeld K. A 4-item measure of depression and anxiety: validation and standardization of the patient health Questionnaire-4 (PHQ-4) in the general population. J Affect Disord. (2010) 122:86–95. doi: 10.1016/j.jad.2009.06.019, PMID: 19616305

[36] Vielsmeier V Marcrum SC Weber FC Langguth B Hintschich C. Audiological effects of COVID-19 infection: results of a standardized interview. Can J Neurol Sci. (2022) 49:623–4. doi: 10.1017/cjn.2021.179, PMID: 34287107 PMC8376841

[37] Davis HE McCorkell L Vogel JM Topol EJ. Long COVID: major findings, mechanisms and recommendations. Nat Rev Microbiol. (2023) 21:133–46. doi: 10.1038/s41579-022-00846-2, PMID: 36639608 PMC9839201

[38] Elmazny A Magdy R Hussein M Elsebaie EH Ali SH Abdel Fattah AM. Neuropsychiatric post-acute sequelae of COVID-19: prevalence, severity, and impact of vaccination. Eur Arch Psychiatry Clin Neurosci. (2023) 273:1349–58. doi: 10.1007/s00406-023-01557-2, PMID: 36707454 PMC9882743

[39] Lopez-Leon S Wegman-Ostrosky T Perelman C Sepulveda R Rebolledo PA Cuapio A. More than 50 long-term effects of COVID-19: a systematic review and meta-analysis. Sci Rep. (2021) 11:16144. doi: 10.1038/s41598-021-95565-8, PMID: 34373540 PMC8352980

[40] Stefanou MI Palaiodimou L Bakola E Smyrnis N Papadopoulou M Paraskevas GP. Neurological manifestations of long-COVID syndrome: a narrative review. Ther Adv Chronic Dis. (2022) 13:20406223221076890. doi: 10.1177/20406223221076890, PMID: 35198136 PMC8859684

[41] Asakura T Kimura T Kurotori I Kenichi K Hori M Hosogawa M. Case–control study of long COVID, Sapporo, Japan. Emerg Infect Dis. (2023) 29:956–66. doi: 10.3201/eid2905.221349, PMID: 37044126 PMC10124665

[42] Iqbal FM Lam K Sounderajah V Clarke JM Ashrafian H Darzi A. Characteristics and predictors of acute and chronic post-COVID syndrome: a systematic review and meta-analysis. EClinicalMedicine. (2021) 36:100899. doi: 10.1016/j.eclinm.2021.100899, PMID: 34036253 PMC8141371

[43] Yong SJ. Long COVID or post-COVID-19 syndrome: putative pathophysiology, risk factors, and treatments. Infect Dis. (2021) 53:737–54. doi: 10.1080/23744235.2021.1924397, PMID: 34024217 PMC8146298

[44] Ahmed SH Waseem S Shaikh TG Qadir NA Siddiqui SA Ullah I. SARS-CoV-2 vaccine-associated-tinnitus: a review. Ann Med Surg. (2022) 75:103293. doi: 10.1016/j.amsu.2022.103293, PMID: 35096388 PMC8788157

[45] Dorney I Bobak L Otteson T Kaelber DC. Prevalence of new-onset tinnitus after COVID-19 vaccination with comparison to other vaccinations. Laryngoscope. (2023) 133:1722–5. doi: 10.1002/lary.30395, PMID: 36098476 PMC9539087

[46] Durai M Searchfield G. Anxiety and depression, personality traits relevant to tinnitus: a scoping review. Int J Audiol. (2016) 55:605–15. doi: 10.1080/14992027.2016.1198966, PMID: 27387463

[47] Trevis KJ McLachlan NM Wilson SJ. A systematic review and meta-analysis of psychological functioning in chronic tinnitus. Clin Psychol Rev. (2018) 60:62–86. doi: 10.1016/j.cpr.2017.12.006, PMID: 29366511

[48] Taylor S Asmundson GJG. Immunization stress-related responses: implications for vaccination hesitancy and vaccination processes during the COVID-19 pandemic. J Anxiety Disord. (2021) 84:102489. doi: 10.1016/j.janxdis.2021.102489, PMID: 34627104 PMC8483981

[49] World Health Organization. Causality assessment of an adverse event following immunization (AEFI): user manual for the revised WHO classification. Geneva: World Health Organization (2019).

[50] Awijen H Ben Zaied Y Nguyen DK. Covid-19 vaccination, fear and anxiety: Evidence from Google search trends. Soc Sci Med. (2022) 297:114820. doi: 10.1016/j.socscimed.2022.114820, PMID: 35183946 PMC8847077

[51] Rozenkrantz L Kube T Bernstein MH Gabrieli JDE. How beliefs about coronavirus disease (COVID) influence COVID-like symptoms? – a longitudinal study. Health Psychol. (2022) 41:519–26. doi: 10.1037/hea0001219, PMID: 35849379

[52] Saunders GH Beukes EW Uus K Armitage CJ Munro KJ. Reporting of auditory symptoms over time: (in)consistencies, expectations and the nocebo effect. Int J Audiol. (2024) 63:1–8. doi: 10.1080/14992027.2022.2163429, PMID: 36779872

[53] Newman CW Jacobson GP Spitzer JB. Development of the tinnitus handicap inventory. Arch Otolaryngol Head Neck Surg. (1996) 122:143–8. doi: 10.1001/archotol.1996.01890140029007 8630207

[54] Vielsmeier V Santiago Stiel R Kwok P Langguth B Schecklmann M. From acute to chronic tinnitus: pilot data on predictors and progression. Front Neurol. (2020) 11:997. doi: 10.3389/fneur.2020.00997, PMID: 33041971 PMC7516990

[55] Lewis RM Jahn KN Parthasarathy A Goedicke WB Polley DB. Audiometric predictors of bothersome tinnitus in a large clinical cohort of adults with sensorineural hearing loss. Otol Neurotol. (2020) 41:e414–21. doi: 10.1097/MAO.0000000000002568, PMID: 32176119 PMC7366362

[56] Gomaa MAM Elmagd MHA Elbadry MM Kader RMA. Depression, anxiety and stress scale in patients with tinnitus and hearing loss. Eur Arch Otorrinolaringol. (2014) 271:2177–84. doi: 10.1007/s00405-013-2715-6, PMID: 24071860

[57] Cohen J Rodgers YM. Long COVID prevalence, disability, and accommodations: analysis across demographic groups. J Occup Rehabil. (2024) 34:335–49. doi: 10.1007/s10926-024-10173-3, PMID: 38388910 PMC11179968

[58] U.S. Department of Health and Human Services. (2021), Guidance on “long COVID” as a disability under the ADA, section 504, and section. Available at: https://www.hhs.gov/civil-rights/for-providers/civil-rights-covid19/guidance-long-covid-disability/index.html (Accessed November 5, 2024).

[59] Hoekstra CEL Wesdorp FM van Zanten GA. Socio-demographic, health, and tinnitus related variables affecting tinnitus severity. Ear Hear. (2014) 35:544–54. doi: 10.1097/AUD.0000000000000045, PMID: 25003528

[60] Strumila R Lengvenytė A Vainutienė V Lesinskas E. The role of questioning environment, personality traits, depressive and anxiety symptoms in tinnitus severity perception. Psychiatry Q. (2017) 88:865–77. doi: 10.1007/s11126-017-9502-2, PMID: 28229347

[61] Kehrle HM Sampaio ALL Granjeiro RC De Oliveira TS Oliveira CACP. Tinnitus annoyance in normal-hearing individuals: correlation with depression and anxiety. Ann Otol Rhinol Laryngol. (2016) 125:185–94. doi: 10.1177/0003489415606445, PMID: 26424781

[62] Bartels H Middel BL van der Laan BF Staal MJ Albers FWJ. The additive effect of co-occurring anxiety and depression on health status, quality of life and coping strategies in help-seeking tinnitus sufferers. Ear Hear. (2008) 29:947–56. doi: 10.1097/AUD.0b013e3181888f83, PMID: 18941410

[63] Jarach CM Lugo A Scala M van den Brandt PA Cederroth CR Odone A. Global prevalence and incidence of tinnitus: a systematic review and meta-analysis. JAMA Neurol. (2022) 79:888–900. doi: 10.1001/jamaneurol.2022.2189, PMID: 35939312 PMC9361184

[64] Haider HF Hoare DJ Costa RFP Potgieter I Kikidis D Lapira A. Pathophysiology, diagnosis and treatment of somatosensory tinnitus: a scoping review. Front Neurosci. (2017) 11:207. doi: 10.3389/fnins.2017.00207, PMID: 28503129 PMC5408030

[65] Saunders GH Beukes E Uus K Armitage CJ Kelly J Munro KJ. Shedding light on SARS-CoV-2, COVID-19, COVID-19 vaccination, and auditory symptoms: causality or spurious conjunction? Front Public Health. (2022) 10:837513. doi: 10.3389/fpubh.2022.837513, PMID: 35296050 PMC8919951

[66] Mennink LM Aalbers MW van Dijk P van Dijk JMC. The role of inflammation in tinnitus: a systematic review and meta-analysis. J Clin Med. (2022) 11:1000. doi: 10.3390/jcm11041000, PMID: 35207270 PMC8878384

[67] Langguth B Kleinjung T Schlee W Vanneste S de Ridder D. Tinnitus guidelines and their evidence base. J Clin Med. (2023) 12:3087. doi: 10.3390/jcm12093087, PMID: 37176527 PMC10178961

