# Supplementary material for: Tinnitus and COVID-19: effect of infection, vaccination, and the pandemic
Source: Front Public Health. 2024 Nov 26;12:1508607. doi: 10.3389/fpubh.2024.1508607 (PMC11629081; doi:10.3389/fpubh.2024.1508607)
Supplement: Supplementary file 1 [file Table_1.docx]

**Supplementary Table S1.** Dissemination channels for the survey.

| **Type** | **Number** |
| --- | --- |
| Tinnitus associations/networks of different countries | 6 |
| Tinnitus support groups | 5 |
| Hearing clinics | 9 |
| Facebook groups | 11 |
| Reddit pages | 2 |
| Instagram pages | 8 |
| Champaign-based newsletters/E-weeks | 3 |
