# Supplementary material for: Tinnitus and COVID-19: effect of infection, vaccination, and the pandemic
Source: Front Public Health. 2024 Nov 26;12:1508607. doi: 10.3389/fpubh.2024.1508607 (PMC11629081; doi:10.3389/fpubh.2024.1508607)
Supplement: Supplementary file 2 [file Table_2.docx]

**Supplementary Table S2.** Multiple linear regression models to predict the Tinnitus Functional Index (TFI) score in different groups after removing outliers and influential observations. Compared to the results of the primary analyses shown in Table 4, the results for most predictor variables did not change significantly. However, hearing loss became a significant predictor variable of the TFI scores in the long COVID group, and both age and gender became non-significant variables of TFI scores in the pre-existing tinnitus group, even though the standardized betas of these predictors did not change much. Notably, the standardized beta of depression increased from 0.133 (Table 4) to 0.251 in the COVID infection group, and the variances explained in the COVID infection and long COVID groups increased from around 27% to > 45% after removing outliers and influential observations, which might reflect the effect of reduced sample sizes.

|  | **COVID infection group**  **(*n* = 104)** | | |  | | **Long COVID group**  **(*n* = 115)** | | |  | **COVID vaccination group**  **(*n* = 509)** | |  | **Pre-existing tinnitus group**  **(*n* = 645)** | |
| --- | --- | --- | --- | --- | --- | --- | --- | --- | --- | --- | --- | --- | --- | --- |
| **Variables** | ***β*** | | ***p*** |  | ***β*** | | ***p*** | |  | ***β*** | ***p*** |  | ***β*** | ***p*** |
| ***Age*** | 0.095 | 0.207 | |  | 0.136 | | | 0.07 |  | 0.14 | **<0.001** |  | 0.07 | 0.053 |
| ***Gender (Male)*** | -0.28 | **<0.001** | |  | | -0.025 | | 0.73 |  | -0.013 | 0.73 |  | -0.068 | 0.051 |
| ***Hearing loss (Yes)*** | 0.181 | **0.017** | |  | | 0.172 | | **0.017** |  | 0.124 | **0.001** |  | 0.163 | **<0.001** |
| ***Hyperacusis (Yes)*** | -0.022 | | 0.768 |  | | 0.212 | | **0.005** |  | 0.106 | **0.007** |  | 0.159 | **<0.001** |
| ***Depression (Yes)*** | 0.251 | | 0.058 |  | | 0.6 | | **<0.001** |  | 0.318 | **<0.001** |  | 0.273 | **<0.001** |
| ***Anxiety (Yes)*** | 0.409 | | **0.002** |  | | 0.042 | | 0.703 |  | 0.245 | **<0.001** |  | 0.226 | **<0.001** |
|  |  | |  |  | |  | |  |  |  |  |  |  |  |
| ***R^2^*** | 0.519 | | |  | | 0.458 | | |  | 0.304 | |  | 0.261 | |
| *β*, Standardized beta  Bold numbers indicate statistical significance. | | | | | | | | | | | | | | |
